# Supplementary material for: Wnt and Nodal asymmetries stratify mouse laterality phenotypes in the absence of node flow
Source: Sci Adv. 2026 Jul 23;12(30):eadx6486. doi: 10.1126/sciadv.adx6486 (PMC13394393; doi:10.1126/sciadv.adx6486)
Supplement: Supplementary file 1 — Figs. S1 to S6 Legends for tables S1, S3 and S7 Tables S2, S4 to S6 Legends for movies S1 to S4 [file sciadv.adx6486_sm.pdf]

Science Advances
AAAS

Supplementary Materials for

Wnt and *Nodal* asymmetries stratify mouse laterality phenotypes in the absence of node flow

Amaia Ochandorena-Saa *et al.*

Corresponding author: Sigolène M. Meilhac, sigolene.meilhac@institutimagine.org

Sci. Adv. **12**, eadx6486 (2026)
DOI: 10.1126/sciadv.adx6486

The PDF file includes:

Figs. S1 to S6
Legends for tables S1, S3 and S7
Tables S2, S4 to S6
Legends for movies S1 to S4

Other Supplementary Material for this manuscript includes the following:

Tables S1, S3 and S7
Movies S1 to S4

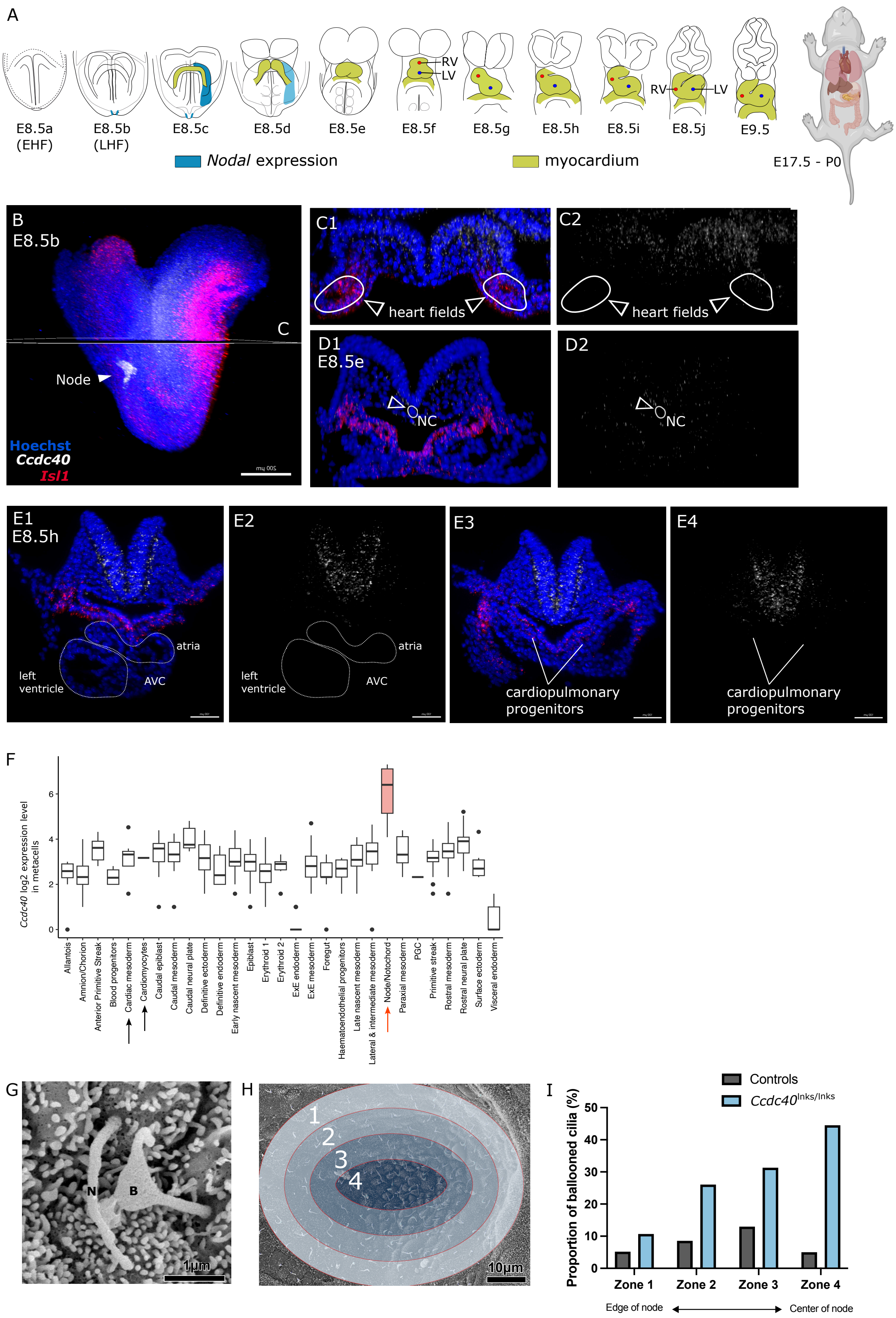
A
E8.5a
(EHF)
E8.5b
(LHF)
E8.5c
E8.5d
E8.5e
E8.5f
E8.5g
E8.5h
E8.5i
E8.5j
E9.5
E17.5 - P0
RV
LV
Nodal expression
myocardium
B
E8.5b
C
Node
Hoechst
Ccdc40
Isl1
200 μm
C1
C2
heart fields
D1
E8.5e
D2
NC
E1
E8.5h
E2
E3
E4
atria
left ventricle
AVC
cardiopulmonary progenitors
F
Ccdc40 log2 expression level in metacells
Allantois
Amnion/Chorion
Anterior Primitive Streak
Blood progenitors
Cardiac mesoderm
Cardiomyocytes
Caudal epiblast
Caudal mesoderm
Caudal neural plate
Definitive ectoderm
Definitive endoderm
Early nascent mesoderm
Epiblast
Erythroid 1
Erythroid 2
ExE endoderm
ExE mesoderm
Foregut
Haematoendothelial progenitors
Late nascent mesoderm
Lateral & intermediate mesoderm
Node/Notochord
Paraxial mesoderm
PGC
Primitive streak
Rostral mesoderm
Rostral neural plate
Surface ectoderm
Visceral endoderm
G
N
B
1μm
H
1
2
3
4
10μm
I
Controls
Ccdc40lnks/lnks
Proportion of ballooned cilia (%)
Zone 1
Zone 2
Zone 3
Zone 4
Edge of node
Center of node

Fig. S1. Expression profile of *Ccdc40* and mapping of abnormal cilia in *Ccdc40* mutant node, related to Fig. 1. (A) Stages of asymmetric heart development (*38*). Myocardium is indicated in yellow, and the position of right and left ventricles (RV, LV) is shown by colored dots. The window of *Nodal* expression is indicated in blue, with expression in the node at E8.5b-c, and expression in the lateral plate mesoderm at E8.5c-d. At E9.5, heart looping is complete, and its shape can be quantified. The cells which have expressed *Nodal* can be traced at this stage with the *Nodal-ASE-lacZ* transgene. In perinatal fetuses (E17.5-P0), visceral organs (heart, lungs, liver, stomach, colon) can be scored for asymmetric features using whole-body Micro-CT. (B-E) Whole-mount in situ hybridisation of *Ccdc40* (white) in wild-type embryos (n=8), relative to *Isl1* (red), used as a marker of cardiac progenitors, at E8.5b (B-C), E8.5e (D) and E8.5h (E). A right lateral view (B) and transverse sections (C-E) are shown. Filled and empty white arrowheads point to high and absent *Ccdc40* expression, respectively. The region of cardiac progenitors in the splanchnic mesoderm (C) and the notochord (NC in panel D) are outlined. (E) During heart looping, *Ccdc40* is neither expressed in the heart (left ventricle, atrioventricular canal (AVC), atria) nor in cardiopulmonary progenitors. (F) Expression of *Ccdc40* in published metacells from E6.5 - E8.25 whole embryos (*94*). The red arrow points to high *Ccdc40* expression in Node/Notochord metacells, and black arrows to basal expression in cardiac metacells. The number of metacells is shown in Table S6. (G) High magnification of cilia morphology, detected by scanning electron microscopy in a *Ccdc40* mutant node at E8.5c. B, ballooned cilium; N, normal cilium. (H) The node was subdivided in four concentric zones. (I) Corresponding quantification of the regionalisation of ballooned cilia. Means are shown over n=2 controls and n=2 mutants. See also Table S7.

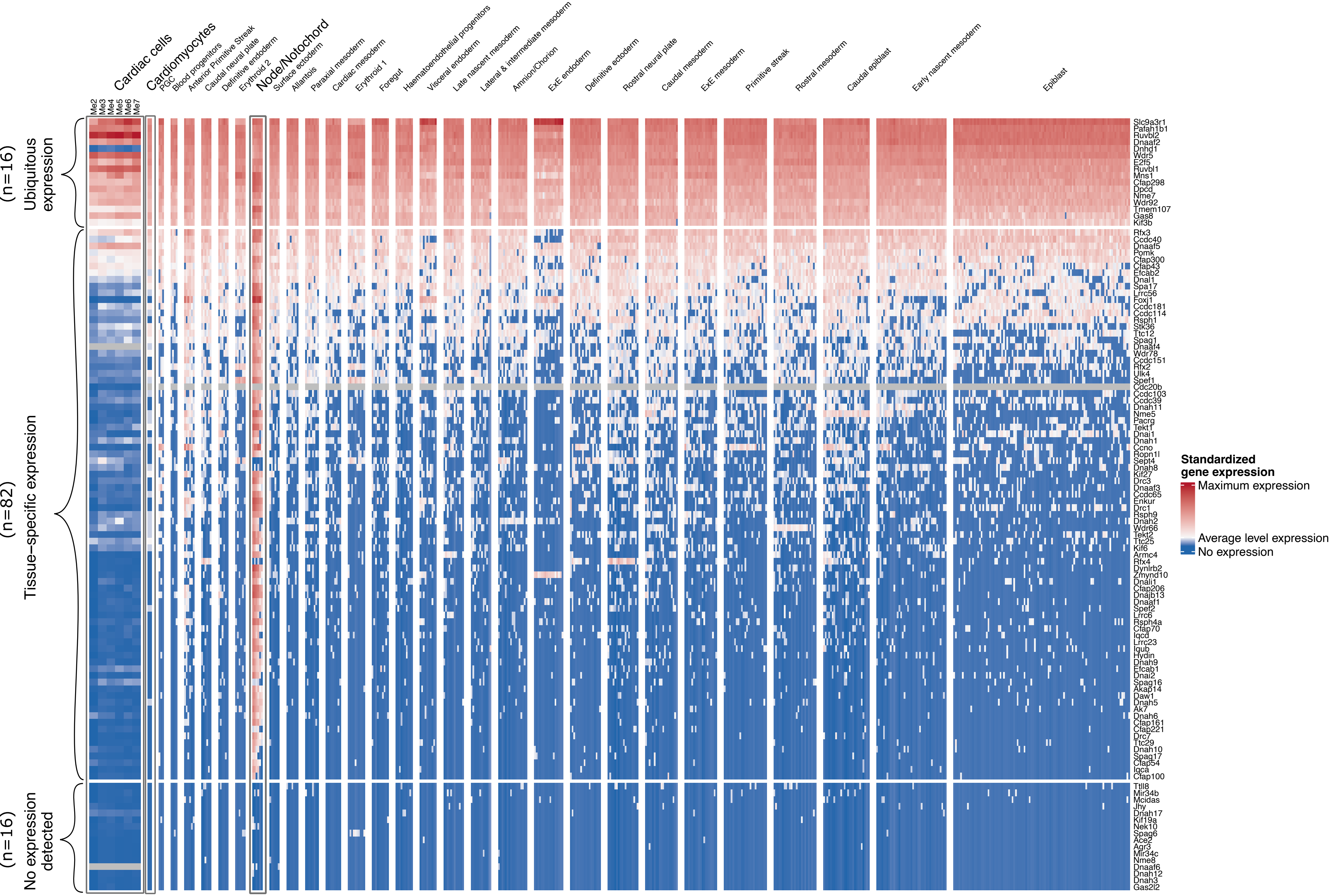
Cardiac cells
Cardiomyocytes
PGC
Blood progenitors
Anterior Primitive Streak
Caudal neural plate
Definitive endoderm
Erythroid 2
Node/Notochord
Surface ectoderm
Allantois
Paraxial mesoderm
Cardiac mesoderm
Erythroid 1
Foregut
Haematoendothelial progenitors
Visceral endoderm
Late nascent mesoderm
Lateral & intermediate mesoderm
Amnion/Chorion
ExE endoderm
Definitive ectoderm
Rostral neural plate
Caudal mesoderm
ExE mesoderm
Primitive streak
Rostral mesoderm
Caudal epiblast
Early nascent mesoderm
Epiblast
Me2
Me3
Me4
Me5
Me6
Me7
(n=16)
Ubiquitous expression
(n=82)
Tissue–specific expression
(n=16)
No expression detected
Slc9a3r1
Pafah1b1
Ruvbl2
Dnaaf2
Dnhd1
Wdr5
E2f5
Ruvbl1
Mns1
Cfap298
Dpcd
Nme7
Wdr92
Tmem107
Gas8
Kif3b
Rfx3
Ccdc40
Dnaaf5
Pomk
Cfap300
Cfap43
Efcab2
Dnal1
Spa17
Lrrc56
Foxj1
Ccdc181
Ccdc114
Rsph1
Stk36
Ttc12
Spag1
Dnaaf4
Wdr78
Ccdc151
Rfx2
Ulk4
Spef1
Cdc20b
Ccdc103
Ccdc39
Dnah11
Nme5
Pacrg
Tekt1
Dnai1
Dnah1
Ccno
Ropn1l
Sept4
Dnah8
Kif27
Drc3
Dnaaf3
Ccdc65
Enkur
Drc1
Rsph9
Dnah2
Wdr66
Tekt2
Ttc25
Kif6
Armc4
Rfx4
Dynlrb2
Zmynd10
Dnali1
Cfap206
Dnajb13
Dnaaf1
Spef2
Lrrc6
Rsph4a
Cfap70
Iqcd
Lrrc23
Iqub
Hydin
Dnah9
Efcab1
Dnai2
Spag16
Akap14
Daw1
Dnah5
Ak7
Dnah6
Cfap161
Cfap221
Drc7
Ttc29
Dnah10
Spag17
Cfap54
Iqca
Cfap100
Ttll8
Mir34b
Mcidas
Jhy
Dnah17
Kif19a
Nek10
Spag6
Ace2
Agr3
Mir34c
Nme8
Dnaaf6
Dnah12
Dnah3
Gas2l2
Standardized gene expression
Maximum expression
Average level expression
No expression

Fig. S2. (separate file) Expression of genes associated with motile cilia in wild-type embryos between E6.5 and E8.5, related to Fig. 1. Heatmap of the expression levels of 114 genes associated with motile cilia. The first 6 columns correspond to mean expression in published single cardiac cells (*39*) (n= 59 Me2 endothelial cells, 713 Me3 cardiomyocytes, 221 Me4 differentiating progenitors, 355 Me5 juxta-cardiac heart field, 65 Me6 differentiating progenitors, 514 Me7 second heart field). The remaining columns correspond to published individual metacells (*94*); the number of metacells is shown in Table S6. Three clusters of expression profile were identified: ubiquitous tissue expression (e.g. *Kif3b*, *Tmem107* which are also expressed in primary cilia), specific expression in Node/Notochord metacells (e.g. *Ccdc40*) and no detected expression at these stages (e.g. *Spag6* required for central pairs of microtubules and *Mcidas, Agr3* specific to multi-ciliated cells). See also Table S7.

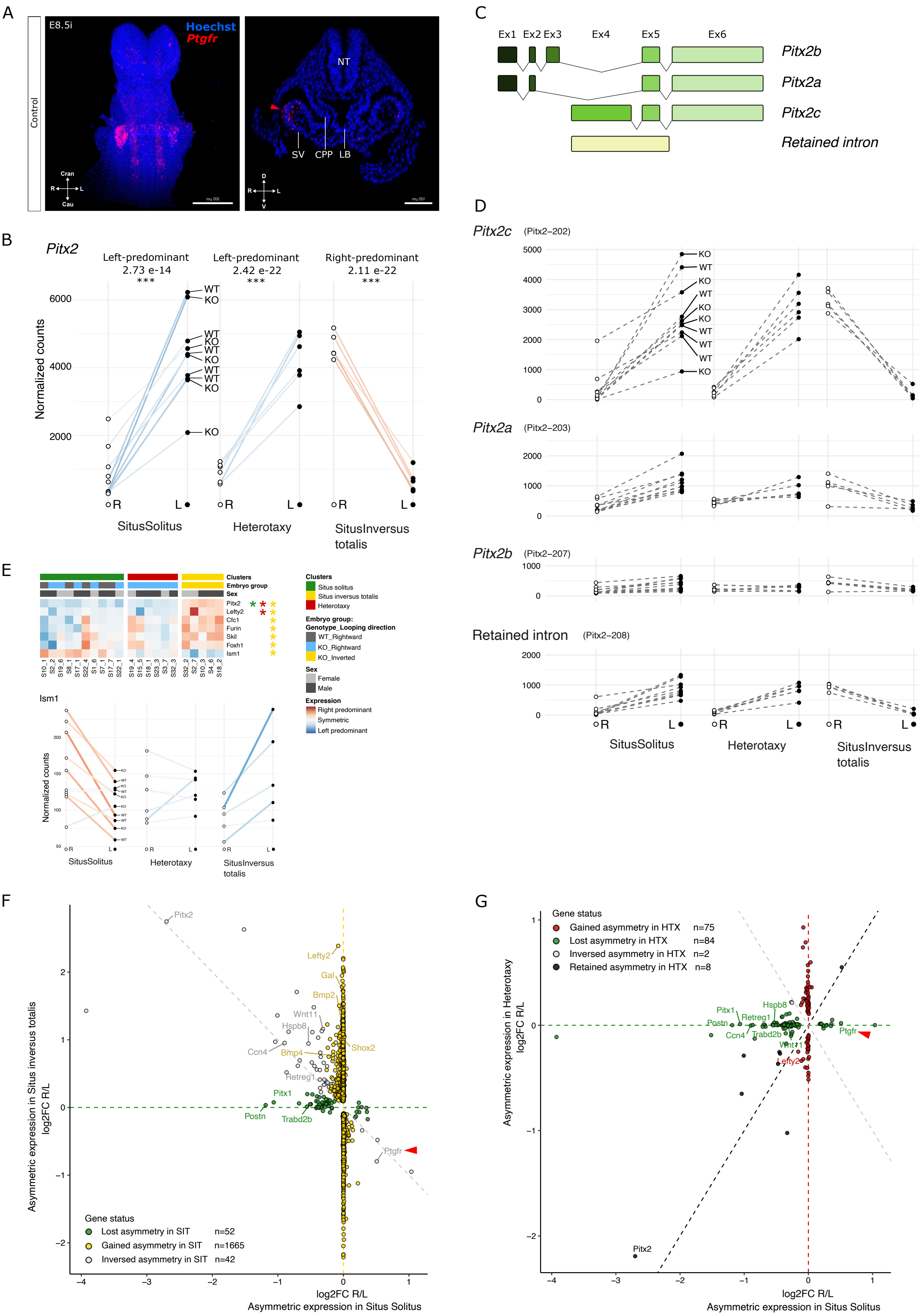
A
E8.5i
Hoechst
Ptgfr
Control
NT
SV
CPP
LB
Cran
Cau
R
L
D
V
B
Pitx2
Left-predominant
2.73 e-14
***
Left-predominant
2.42 e-22
***
Right-predominant
2.11 e-22
***
Normalized counts
SitusSolitus
Heterotaxy
SitusInversus totalis
C
Ex1 Ex2 Ex3 Ex4 Ex5 Ex6
Pitx2b
Pitx2a
Pitx2c
Retained intron
D
Pitx2c (Pitx2-202)
Pitx2a (Pitx2-203)
Pitx2b (Pitx2-207)
Retained intron (Pitx2-208)
E
Clusters
Embryo group
Sex
Situs solitus
Situs inversus totalis
Heterotaxy
Embryo group: Genotype_Looping direction
WT_Rightward
KO_Rightward
KO_Inverted
Female
Male
Expression
Right predominant
Symmetric
Left predominant
Pitx2
Lefty2
Cfc1
Furin
Skil
Foxh1
Ism1
F
Gene status
Lost asymmetry in SIT n=52
Gained asymmetry in SIT n=1665
Inversed asymmetry in SIT n=42
Asymmetric expression in Situs inversus totalis log2FC R/L
log2FC R/L
Asymmetric expression in Situs Solitus
G
Gene status
Gained asymmetry in HTX n=75
Lost asymmetry in HTX n=84
Inversed asymmetry in HTX n=2
Retained asymmetry in HTX n=8
Asymmetric expression in Heterotaxy log2FC R/L
log2FC R/L
Asymmetric expression in Situs Solitus

Fig. S3. Additional transcriptomic analyses in *Ccdc40* mutants at E8.5i, related to Fig. 7.
(A) Fluorescent whole-mount in situ hybridisation of *Ptgfr* (red) in a wild-type embryo (n=4), validating the right-sided asymmetry identified by transcriptomics. The red arrowhead points to expression in the sinus venosus (SV). (B-D) Plot of *Pitx2* expression levels by transcriptomics in the indicated embryo clusters. Aggregated counts are shown (B), as well as counts specific to each *Pitx2* isoform (D), as schematised in (C). The adjusted p-values of right over left levels are shown in (B). Isoform names in brackets are from Ensembl. (E) Heatmap of asymmetric expression of NODAL pathway genes. Asterisks indicate significance per cluster (adjusted p-val). The genotype, sex, heart looping direction and transcriptomic cluster of embryos is colour-coded as indicated. The plot of *Ism1* expression levels is shown below. (F-G) Plots of comparative asymmetric gene expression in the three embryo clusters. Significant changes in gene asymmetry in situs inversus totalis (F) and heterotaxy (G) compared to situs solitus cluster is colour-coded as indicated. The significant asymmetry of *Ptgfr* is indicated by a red arrowhead. CPP, cardiopulmonary progenitors; Cran, cranial; Cau, caudal; D, dorsal; HTX, heterotaxy; KO, knock-out ($Ccdc40^{lnks/lnks}$); L, left; LB, lung bud; NT, neural tube; R, right; SIT, situs inversus totalis; V, ventral ; WT, wild-type.

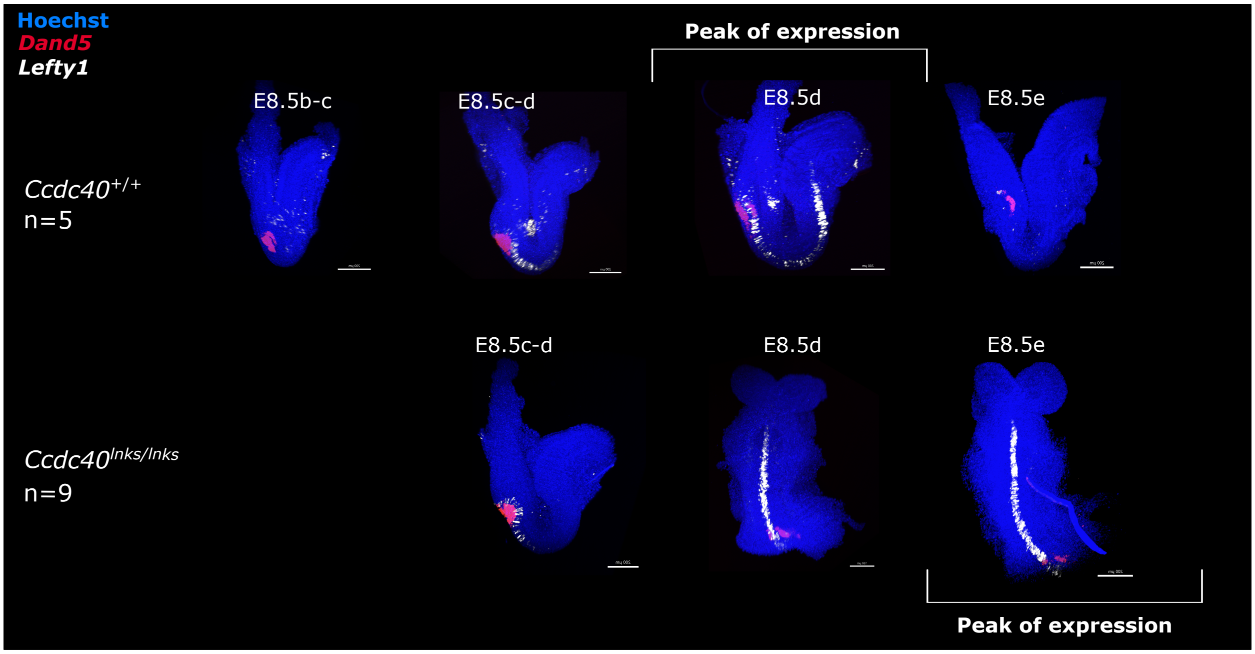

Fig. S4. *Lefty1* expression is delayed in *Ccdc40* mutant embryos, related to Figs. 6-7. Fluorescent whole-mount in situ hybridisation in *Ccdc40* mutants and wild-type littermates, at the stages indicated. *Dand5* (red) is expressed in the node. *Lefty1* expression (white) in the midline turns on at E8.5c and peaks at E8.5d in wild-types, while it is detected slightly later and peaks at E8.5e in mutants, indicating an overall delay. *Lefty1* is also expressed in the lateral plate mesoderm, at a much lower level which cannot be shown without saturation of the midline signal. It is reported in transverse sections in Fig. 6.

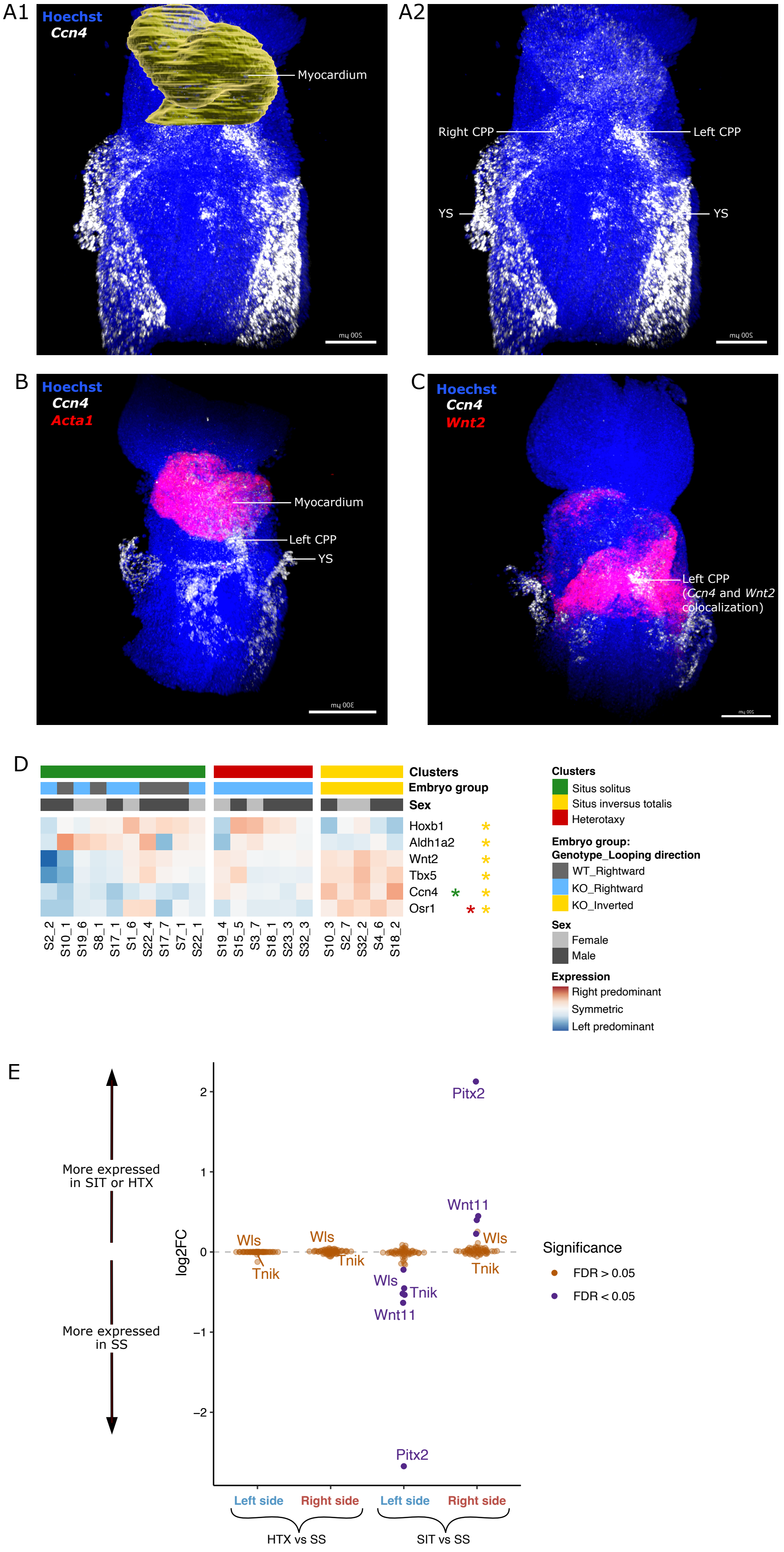

A1
Hoechst
Ccn4
Myocardium
A2
Right CPP
Left CPP
YS
YS
B
Hoechst
Ccn4
Acta1
Myocardium
Left CPP
YS
C
Hoechst
Ccn4
Wnt2
Left CPP
(Ccn4 and Wnt2 colocalization)
D
Clusters
Embryo group
Sex
Hoxb1
Aldh1a2
Wnt2
Tbx5
Ccn4
Osr1
S2_2
S10_1
S19_6
S8_1
S17_1
S1_6
S22_4
S17_7
S7_1
S22_1
S19_4
S15_5
S3_7
S18_1
S23_3
S32_3
S10_3
S2_7
S32_2
S4_6
S18_2
Clusters
Situs solitus
Situs inversus totalis
Heterotaxy
Embryo group:
Genotype_Looping direction
WT_Rightward
KO_Rightward
KO_Inverted
Sex
Female
Male
Expression
Right predominant
Symmetric
Left predominant
E
More expressed in SIT or HTX
More expressed in SS
log2FC
2
1
0
−1
−2
Pitx2
Wnt11
Wls
Tnik
Wls
Tnik
Wls
Tnik
Wls
Tnik
Wnt11
Pitx2
Left side
Right side
Left side
Right side
HTX vs SS
SIT vs SS
Significance
FDR > 0.05
FDR < 0.05

Fig. S5. Expression profile of *Ccn4* and transcriptomic profile of markers of cardiopulmonary progenitors, related to Fig. 8. (A-C) Whole-mount in situ hybridisation of *Ccn4* (white) in wild-type embryos at E8.5i, seen in a ventral view. The heart tube is segmented in A1 (yellow). Co-staining (red) with a marker of cardiomyocytes (*Acta1*, B) or a marker of cardiopulmonary progenitors (*Wnt2*, C) is shown. (D) Heatmap of expression asymmetry of genes marking cardiopulmonary progenitors, detected by transcriptomics. The genotype, sex, heart looping direction and transcriptomic cluster of embryos is colour-coded as indicated. Asterisks indicate significance per cluster (adjusted p-val). (E) Plot of changes in expression levels of WNT pathway genes (n=56) comparing one morphological side between two embryo clusters, as indicated below. CPP, cardiopulmonary progenitors; FDR, false discovery rate; HTX, heterotaxy; SIT, situs inversus totalis; SS, situs solitus; YS, yolk sac.

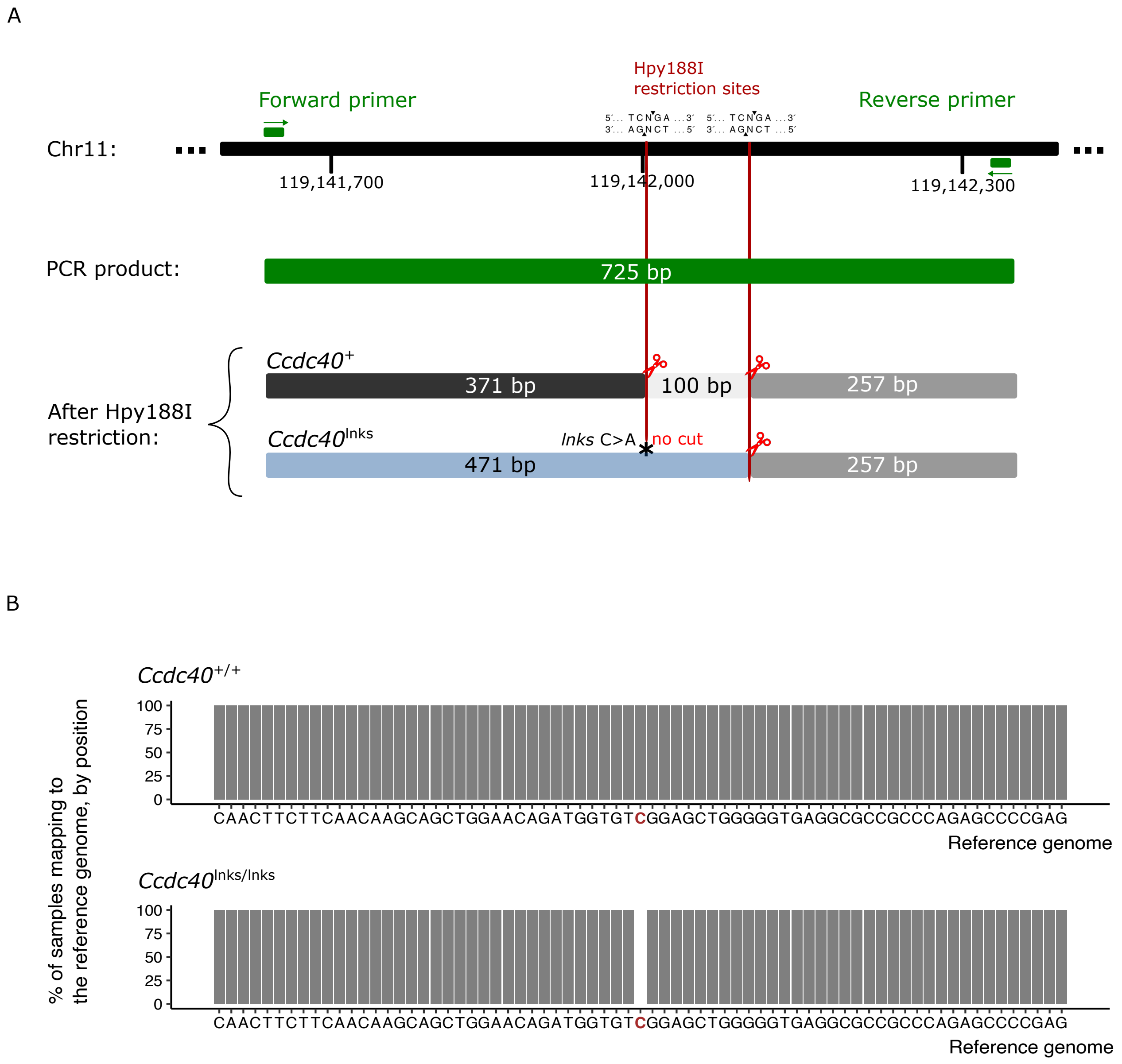

Fig. S6. Genotyping of *Ccdc40* mutants, related to Methods. (A) Schematic representation of the *Ccdc40* locus, showing the localisation of the ENU-induced nonsense point mutation in the *Ccdc40^{lnks}* allele. Primers and restriction sites used for genotyping are mapped according to the GRCm39 nomenclature, and the PCR products before and after restriction are shown. (B) Validation of the genotypes of samples used for transcriptomics, showing sample sequence mapping to the reference genome.

Table S1. (separate file) Individual phenotypes of *Ccdc40* mutants at birth, related to Fig. 2. Phenotypes were evaluated by micro-CT and HREM. The 3-letter segmental nomenclature (*89*) of congenital heart defects is provided in columns O-Q. The phenotypes are colour-coded as normal situs solitus (green), situs inversus (yellow) or abnormal (orange). Grey indicates mild anomalies not diagnostic of laterality defects. A, situs ambiguous (column P) or A-malposition (column R); D, D-Loop (column Q) or D-malposition (column R); DORV, double-outlet right ventricle; I, situs inversus; L, L-Loop (column Q) or L-malposition (column R); S, situs solitus; TAPVR, total anomalous pulmonary venous return; TGA, transposition of the great arteries; VSD, ventricular septal defect.

Table S2. Spectrum of Tg *Nodal-ASE-lacZ* patterns in *Ccdc40* mutant hearts at E9.5, related to Fig. 5. In one sample with inverted heart looping (*), the Tg *Nodal-ASE-lacZ* pattern was too faint to be analysed. AVC, atrioventricular canal; CPP, cardiopulmonary progenitors; DPW, dorsal pericardial wall; L, left; LV, left ventricle; OFT, outflow tract; R, right; RV, right ventricle.

		Xgal-positive pattern (E9.5)						
Genotype	Looping direction	Signal in OFT	Signal in AVC	Signal in atrium facing LV	Signal in atrium facing RV	Signal in DPW	CPP signal	Posterior LPM signal
$Ccdc40^{+/+}$; Tg Nodal-ASE-lacZ	Rightward 12/12 (100%)	Inferior-left	Superior-right	Dorsal	None	Left	Left	Left
$Ccdc40^{lnks/lnks}$; Tg Nodal-ASE-lacZ	Rightward 17/23 (74%)	Inferior-left	Superior-right (13/17), Inferior-right (3/17), Absent (1/17)	Dorsal	None (14/17) Dorsal+ventral (2/17), Ventral (1/17)	Left (15/17), None (2/17)	Left (13/17), Bilateral R>L (2/17), Bilateral R=L (1/17), None (1/17)	Left (11/17), Bilateral R=L (4/17), Bilateral R>L (2/17)
	Inverted 6*/23 (26%)	Inferior-right (5/5)	Inferior-left (1/5), Superior-left (4/5)	Dorsal (5/5)	Dorsal+ventral (1/5), Ventral (2/5), None (2/5)	None (1/5), Right (4/5)	Bilateral R=L (2/5), Right (3/5)	Bilateral R=L (4/5), Bilateral R>L (1/5)

Table S3. (separate file) Individual phenotypes of *Ccdc40* mutants and controls at E9.5, related to Fig. 5. Input parameters for unsupervised clustering of embryos are shown. Geometrical parameters were extracted from 3D segmentations of the heart tube. Descriptors of the Tg *Nodal-ASE-lacZ* patterns are provided, when available (blue columns). The phenotypic group, as an output of unsupervised clustering, is labelled in the last column. AVC, atrioventricular canal; bif, bifurcation of the tube from the atrioventricular canal to the atria; CPP, cardiopulmonary progenitors; doft, distal outflow tract; DPW, dorsal pericardial wall; IVS, interventricular septum; L, left; LA, left atrium; LPM, lateral plate mesoderm; LV, left ventricle; OFT, outflow tract; R, right; RA, right atrium; RV, right ventricle.

Table S4. Asymmetric pattern of *Dand5* and *Lefty1* in *Ccdc40*$^{lnks/lnks}$ mutant embryos, related to Fig. 6. The asymmetry of *Dand5* is quantified and coloured when significantly asymmetric. The colour-code reflects patterns associated with situs solitus (green), heterotaxy (red) and situs inversus totalis (yellow). L, left; LPM, lateral plate mesoderm; R, right.

Embryo ID	Stage	***Dand5* crown (Log2 L/R)**	***Lefty1* floorplate**	***Lefty1* LPM**
C805	E8.5c-d	0.43	Center	none
C535	E8.5d	0.62	Left	none
C679	E8.5d	0.11	Right	Bilateral
C744	E8.5d-e	0.47	Center	Left
C466	E8.5e	0.05	Left	Left
C459	E8.5e	0.91	Right	Right

Table S5. List of differentially expressed genes belonging to Wnt GO terms, in transcriptomic clusters at E8.5i, related to Fig. 7.

Ensembl ID	Gene name	Wnt GO term that the gene belongs to<br>(if it belongs to various Wnt GO lists, only one is shown)
ENSMUSG00000031486	Adgra2	GO:0016055_Wnt_signaling_pathway
ENSMUSG00000032531	Amotl2	GO:0016055_Wnt_signaling_pathway
ENSMUSG00000000142	Axin2	GO:0016055_Wnt_signaling_pathway
ENSMUSG00000023055	Calcoco1	GO:0016055_Wnt_signaling_pathway
ENSMUSG00000005124	Ccn4	GO:0016055_Wnt_signaling_pathway
ENSMUSG00000070348	Ccnd1	GO:0016055_Wnt_signaling_pathway
ENSMUSG00000005087	Cd44	GO:0016055_Wnt_signaling_pathway
ENSMUSG00000028926	Cdk14	GO:0016055_Wnt_signaling_pathway
ENSMUSG00000068740	Celsr2	GO:0016055_Wnt_signaling_pathway
ENSMUSG00000034574	Daam1	GO:0016055_Wnt_signaling_pathway
ENSMUSG00000040260	Daam2	GO:0016055_Wnt_signaling_pathway
ENSMUSG00000000787	Ddx3x	GO:0016055_Wnt_signaling_pathway
ENSMUSG00000032064	Dixdc1	GO:0016055_Wnt_signaling_pathway
ENSMUSG00000024868	Dkk1	GO:0016055_Wnt_signaling_pathway
ENSMUSG00000030792	Dkkl1	GO:0060070_canonical_Wnt_signaling_pathway
ENSMUSG00000037370	Enpp1	GO:0016055_Wnt_signaling_pathway
ENSMUSG00000006311	Etv2	GO:0016055_Wnt_signaling_pathway
ENSMUSG00000037712	Fermt2	GO:0016055_Wnt_signaling_pathway
ENSMUSG00000078302	Foxd1	GO:0060070_canonical_Wnt_signaling_pathway
ENSMUSG00000067199	Frat1	GO:0060070_canonical_Wnt_signaling_pathway
ENSMUSG00000081683	Fzd10	GO:0016055_Wnt_signaling_pathway
ENSMUSG00000049791	Fzd4	GO:0016055_Wnt_signaling_pathway
ENSMUSG00000041075	Fzd7	GO:0016055_Wnt_signaling_pathway
ENSMUSG00000002996	Hbp1	GO:0016055_Wnt_signaling_pathway
ENSMUSG00000024986	Hhex	GO:0016055_Wnt_signaling_pathway
ENSMUSG00000001552	Jup	GO:0060070_canonical_Wnt_signaling_pathway
ENSMUSG00000028649	Macf1	GO:0016055_Wnt_signaling_pathway
ENSMUSG00000035158	Mitf	GO:0016055_Wnt_signaling_pathway
ENSMUSG00000004558	Ndrg2	GO:0016055_Wnt_signaling_pathway
ENSMUSG00000005397	Nid1	GO:0060070_canonical_Wnt_signaling_pathway
ENSMUSG00000021567	Nkd2	GO:0016055_Wnt_signaling_pathway
ENSMUSG00000070526	Peg12	GO:0060070_canonical_Wnt_signaling_pathway
ENSMUSG00000028023	Pitx2	GO:0016055_Wnt_signaling_pathway
ENSMUSG00000031169	Porcn	GO:0016055_Wnt_signaling_pathway
ENSMUSG00000036158	Prickle1	GO:0016055_Wnt_signaling_pathway
ENSMUSG00000028518	Prkaa2	GO:0016055_Wnt_signaling_pathway
ENSMUSG00000034177	Rnf43	GO:0016055_Wnt_signaling_pathway
ENSMUSG00000019880	Rspo3	GO:0016055_Wnt_signaling_pathway
ENSMUSG00000031548	Sfrp1	GO:0016055_Wnt_signaling_pathway
ENSMUSG00000027996	Sfrp2	GO:0016055_Wnt_signaling_pathway
ENSMUSG00000018822	Sfrp5	GO:0016055_Wnt_signaling_pathway
ENSMUSG00000001494	Sost	GO:0016055_Wnt_signaling_pathway
ENSMUSG00000074637	Sox2	GO:0016055_Wnt_signaling_pathway
ENSMUSG00000000567	Sox9	GO:0060070_canonical_Wnt_signaling_pathway
ENSMUSG00000030782	Tgfb1i1	GO:0016055_Wnt_signaling_pathway
ENSMUSG00000045377	Tmem88	GO:0016055_Wnt_signaling_pathway
ENSMUSG00000027692	Tnik	GO:0016055_Wnt_signaling_pathway
ENSMUSG00000031529	Tnks	GO:0016055_Wnt_signaling_pathway
ENSMUSG00000070867	Trabd2b	GO:0016055_Wnt_signaling_pathway
ENSMUSG00000026556	Vangl2	GO:0035567_non-canonical_Wnt_signaling_pathway
ENSMUSG00000028173	Wls	GO:0016055_Wnt_signaling_pathway
ENSMUSG00000015957	Wnt11	GO:0016055_Wnt_signaling_pathway
ENSMUSG00000029671	Wnt16	GO:0016055_Wnt_signaling_pathway
ENSMUSG00000010797	Wnt2	GO:0016055_Wnt_signaling_pathway
ENSMUSG00000033227	Wnt6	GO:0016055_Wnt_signaling_pathway
ENSMUSG00000030093	Wnt7a	GO:0016055_Wnt_signaling_pathway

Table S6. List of primers used for genotyping, related to Methods.

Primer name	Primer sequence (5′-3′)	Source
Ccdc40 forward primer	GATGCAGGGCCAGTTAGCTT	This paper
Ccdc40 reverse primer	CAGCGTCTCCTGCTGAACCT	This paper
Nodal-ASE-LacZ forward primer	GGCGACTTCCAGTTCAACATC	This paper
Nodal-ASE-LacZ reverse primer	GGCTCAACATGTACGCCAGA	This paper
Sex genotyping forward primer	GATGATTTGAGTGGAAATGTGAGGTA	McFarlane et al, 2013
Sex genotyping reverse primer	CTTATGTTTATACGGCATGCACCATGTA	McFarlane et al, 2013

Table S7. (separate file) Numerical data used to generate figure graphs along with statistical tests, related to Fig. 1-3, 6, 8 and S1.

Movie S1. Examples of live-imaging of the node flow, related to Fig. 1. The first sequence is a wild-type node at E8.5b and the second sequence a *Ccdc40* mutant node at E8.5b, in which fluorescent beads have been added. The associated flow map by Particle Image Velocimetry is shown.

Movie S2. Example of laterality phenotyping at E17.5, related to Fig. 2. The first sequence is a micro-CT scan of visceral organs in situ in a wild-type fetus. 3D rendering of the scan showing the external contour of the thoracic and abdominal cavities is provided, followed by serial frontal sections. The second sequence is a 3D image of the cardiac anatomy by HREM. 3D rendering showing the external heart contour is provided, followed by serial frontal sections. Some bright artefactual aggregates of the contrast agent are seen. Examples of normal laterality, i.e. situs solitus, abnormal laterality, with situs inversus totalis, and abnormal laterality, with heterotaxy and left isomerism, in *Ccdc40* mutants follow, with the same imaging modalities. Individual phenotypes are described in Table S1.

Movie S3. Example of 3D imaging by HREM and heart segmentation at E9.5, related to Fig. 3. The first sequence is a 3D rendering showing the external contour of a wild-type embryo, followed by serial transverse sections. Whole mount in situ hybridisation of *Wnt11*, a marker of the outflow tract, and *Bmp2*, a marker of the atrioventricular canal and left atrium, appears in blue, whereas histology is in grey. In the third sequence, segmented cardiac regions are shown: outflow tract (green), right ventricle (red), left ventricle (blue), atrio-ventricular canal (yellow), left atrium (orange), right atrium (pink). The notochord (turquoise) is used as a reference cranio-caudal axis. In the final sequence, the isolated heart is shown, with landmarks used for quantification (white): the tube circumference at the exit of the outflow tract and in the atrio-ventricular canal at the bifurcation to left/right atria, as well as cranio-caudal and dorso-ventral axes.

Movie S4. Example of 3D imaging by HREM of a *Ccdc40$^{+/+}$; Tg Nodal-ASE-lacZ* embryo at E9.5, related to Fig. 5. The first sequence is a 3D rendering showing the external contour of the embryo, as well as β-galactosidase staining in red, followed by serial transverse sections. In the next sequence, the segmented β-galactosidase staining is shown whole mount. Finally, the staining is shown within the segmented heart. In the final sequence, cardiac regions are colour-coded (outflow tract in green, right ventricle in red, left ventricle in blue, atrioventricular canal in yellow, left atrium in orange, right atrium in pink). The *Nodal-ASE-lacZ* transgene is used to trace contribution to the embryo of cells that have transiently expressed left-sided *Nodal* at E8.5c–d. *Nodal*-expressing cells thus contribute to left lateral plate mesoderm, the anterior forelimbs, second heart field progenitors in the dorsal pericardial wall, cardiopulmonary progenitors, myocardium in the inner curvature, including the left outflow tract, the superior atrioventricular canal and the dorsal left atrium.
